# The effect of lacosamide on calcitonin gene-related peptide serum level in episodic migraine patients: a randomized, controlled trial

**DOI:** 10.1007/s13760-024-02499-9

**Published:** 2024-03-19

**Authors:** Shimaa Elgamal, Sherihan Rezk Ahmed, Mohamed M. Nahas, Shimaa R. Hendawy, Osama Elshafei, Mohamed G. Zeinhom

**Affiliations:** 1 Neurology Department, Faculty of Medicine, Kafr El-Sheikh University, Elgeish Street, Kafr El-Sheikh, Egypt; 2 Clinical Pathology Department, Faculty of Medicine, Mansoura University, Al Korneish Street, Mansoura, Egypt; 3 Neurology Department, Faculty of Medicine, Mansoura University, Al Korneish Street, Mansoura, Egypt

**Keywords:** Episodic migraine, Calcitonin gene-related peptide, Egypt, Lacosamide

## Abstract

**Background:**

Migraine affects 11–15% of people worldwide, and the calcitonin gene-related peptide (CGRP) is released during the migraine attack, producing pulsating pain of migraine. Also, lacosamide reacts with collapsin-response mediator protein 2, preventing its phosphorylation and leading to the inhibition of CGRP release in the trigeminal system.

**Objective:**

The primary outcome was the difference in the serum level of CGRP-LI after three months of treatment with either lacosamide and ibuprofen or ibuprofen alone in episodic migraine patients. The secondary outcomes were assessing safety and efficacy of lacosamide in episodic migraine patients.

**Methods:**

We conducted an open-label randomized controlled trial on episodic migraine patients aged 10–55 years diagnosed according to (ICHD-3) in Kafr El-Sheikh University Hospital, Egypt. We assessed serum levels of CGRP-LI before and three months after treatment in our two groups, the lacosamide, and the control groups. We also assessed the side effects of treatment in each group, the percentage of patients who achieved ≥ 50% reduction in the migraine monthly days (MMD) frequency and the percentage of patients who achieved pain freedom within 2 h in ≥ 4 of 5 attacks in each group.

**Results:**

200 episodic migraine patients completed the study. There was a statistically significantly higher reduction in the serum CGRP-LI level in the lacosamide group compared with the control group. In addition, lacosamide was well tolerated by patients. Also, the lacosamide group had statistically significant higher percentage of patients who achieved ≥ 50% reduction in the migraine monthly days (MMD) frequency and pain freedom within two hours in ≥ 4 of 5 attacks with *P*-values 0.002, 0.02 respectively.

**Conclusion:**

The daily use of lacosamide 50 mg Bid for three months in episodic migraine patients was associated with a significant reduction in serum CGRP-LI, better clinical outcomes regarding frequency and duration of migraine attacks, and was well tolerated by patients. These results were derived from an open-label pilot study that needed to be thoroughly investigated by a large-scale, randomized, double-blinded, placebo-controlled study.

*Trial registration:* We registered our trial on ClinicalTrials.gov, named after "The Lacosamide's Effect on Calcitonin Gene-related Peptide in Migraine Patients," and with a clinical trial number (NCT05632133)—August 8, 2023.

## Introduction

Migraine affects 11–15% of people worldwide, and it is well known that recurrent mild to severe pain and other autonomic symptoms are the hallmarks of migraines [1].

The neurovascular theory is a pathophysiological mechanism of migraines that is most widely recognized. This theory's mainstay is that, in genetically susceptible people, alterations in the nociceptive inputs from the raphe and locus coeruleus nuclei in the brainstem or a cortical spreading depression stimulate trigeminovascular system producing migraine pain and associated symptoms [2].

CGRP is one of the most significant peptides released during the migraine attack due to activation of the trigeminovascular system. It produces vasodilatation and inflammation in leptomeningeal and extracranial vessels, which causes the pulsating pain associated with migraine [2].

Serum CGRP values are increased at the time of an attack in patients who suffer from episodic migraines, while in patients who suffer from chronic migraines, the serum CGRP values rise even in between attacks [3].

In 2010, Hansen and colleagues found that the intravenous administration of CGRP in patients known to have migraines caused a migraine-like headache in about 60% of the patients [4].

The tailored amino acid compound lacosamide (R-2-acetamido-N-benzyl-3-methoxypropionamide) has two distinct mechanisms: the first one is the interaction with CRMP2, and the second one is delaying inhibition of voltage-gated calcium channels (VGSCs) [5].

Lacosamide increases the slow inactivation of VGSCs by making the slow inactivation curve potential more hyperpolarized. As a result, depolarization is prolonged, and many other membrane potentials move to the slow inactivation state. Also, fewer neurons become depolarized, and in the end, this might reduce the spread of an excitatory focus [6].

It has been postulated that Lacosamide reacts with CRMP-2 and prevents its phosphorylation leading to the inhibition of CGRP release in the trigeminal system [7].

In 2019, Yuan and colleagues found that lacosamide had a potential efficacy in migraine treatment. But till now, the clinical investigation has yet to be started [8].

In our trial, we aimed to study the effect of lacosamide on CGRP levels in episodic migraine patients to investigate its possible role in migraine management.

## Materials and methods

### Sample size

We screened the 1218 headache patients who suffered from headaches and sought medical advice in Kafr El-Sheik University Hospital from June 2022 to June 2023. Five hundred forty patients had migraine (380 patients had episodic migraine, 160 had chronic migraine). According to ICHD-3 [9], 200 patients had episodic migraine, met our inclusion criteria, and agreed to participate in our trial after getting written informed consent from the patients or their first-of-kin relatives and after approval from the ethical committee of the faculty of medicine at Kafr El-Sheik University.

We used G.power software to calculate the power of our study based on the mean and the standard deviation of the absolute reduction in CGRP-LI in each group, which was 2.8 ± 1.07 in the lacosamide group and 1.73 ± 1.76 in the control group, 95% two-sided confidence level, alpha error of 5%, effect size of 0.73, and 5% loss to follow-up rate. The power of our study was 99%.

We used a web-based centralized blocked randomization plan to allocate 200 episodic migraine in a one-to-one ratio to receive either lacosamide 50 mg Bid and ibuprofen 200–400 mg on acute attacks only or ibuprofen 200–400 mg on acute attacks only. Still, all clinical investigators were blind to the block size of the randomization plan, but the patients were aware of the treatment used in the study.

Our trial had two groups: the lacosamide group, which included 100 episodic migraines (those with migraines who have less than 15 headache days per month) following ICHD-3 [9] and received fixed daily dose of lacosamide (50 mg Bid) and ibuprofen 200–400 mg only during acute migraine attack [10] for three months, and the control group which included 100 episodic migraine patients who were diagnosed following ICHD-3 [9] and received ibuprofen 200–400 mg only during acute migraine attack [10] for three months, and we identified their migraine features (disease duration, migraine monthly days, attack duration, pain intensity assessed by visual analogic scale) with the help of a questionnaire.

Our investigation was explicitly intended to serve as a pilot study to investigate the preliminary effect of lacosamide on the serum level of CGRP-LI to identify its possible role in episodic migraine management, as well as to determine whether it would be feasible to proceed with a large-scale randomized clinical trial that would be sufficiently powered to evaluate the safety and effectiveness of lacosamide in episodic migraine sufferers.

#### Inclusion criteria:

Our trial enrolled patients aged 10–55 years suffering from episodic migraines following ICHD-3. [11], and all the patients did not receive any migraine preventive treatment or triptans in the last month.

#### Exclusion criteria

We ruled out patients with major neurological conditions, such as (primary headaches other than episodic migraine, stroke, epilepsy, and brain tumors, as well as patients with significant systemic diseases, such as malignancy, liver cell failure, renal failure, patients with MRI contraindications, patients who received any migraine preventive treatment or triptans in the last month pregnant, and lactating patients, patients who had cardiovascular diseases as heart failure, ischemic heart disease, heart block, and atrial fibrillation, and those who had lacosamide hypersensitivity or contraindications.

### Study procedures

We screened 1218 patients, and all underwent clinical neurological and general physical examinations. Migraine history, type, and associated phenotypic features were established. We measured blood pressure on three different occasions and did laboratory tests, including (renal functions, coagulation profile, fasting, postprandial blood sugar, liver functions, and complete blood count). We excluded 1018 patients; 200 patients underwent randomization, received at least one treatment dose, and were included in the analysis.

One hundred twenty participants in our trial were females; 56 patients had migraine with aura, the median monthly migraine days was seven days, the median migraine attack duration was six hours, the median duration of migraine was eight months, 163 patients had photophobia in association to migraine, 158 had phonophobia in association to migraine, 157 had nausea associated with migraine, 137 had dizziness with migraine, and 71 patients suffered from vomiting concomitant with migraine.

All the patients who fulfilled the inclusion criteria were invited to the hospital before starting treatment and three months after treatment to analyze the serum level of CGRP-like immunoreactivity (CGRP-LI).

Blood samples were collected from the antecubital vein at both time points while subjects rested in a sitting position. The blood was then allowed to clot and was centrifuged at room temperature for 20 min at 622 *g*/2000 rpm to separate serum. Samples were then immediately analyzed. For radioimmunoassay (RIA), a commercial kit (CGRP (Human)—RIA Kit (Phoenix Pharmaceuticals, Burlingame, California, United States), detection range 0.53–660 pmol/l), was used following manufacturer's instructions to measure CGRP-like immunoreactivity (CGRP-LI) levels. All samples were analyzed in the same laboratory, under the same environmental conditions, and with the same batch for samples from different patients and study days to avoid a possible batch effect. Samples with values outside the detection range were set on the limits of the detection range. Biochemical assays were performed by an experienced lab technician blinded to the patient identity, study day, and treatment effect of lacosamide.

After three months of treatment, we assessed the difference in CGRP-LI levels in the two groups to evaluate the effect of lacosamide on CGRP-LI levels; also, we assessed the safety of lacosamide by assessing the different adverse effects through open‐ended patient interviews in the two groups, in addition, we evaluated the percentage of patients who achieved ≥ 50% reduction in the migraine monthly days (MMD) frequency compared to the baseline frequency [12], and the percentage of patients who achieved pain freedom within two hours in ≥ 4 of 5 attacks [13], Although migraine is a chronic disease, in our study, we assessed the lacosamide effects on CGRP-LI level and the clinical outcomes after three months [14–17] as our trial was a pilot one aimed mainly to investigate the preliminary effect of lacosamide on the serum level of CGRP-LI and evaluate the feasibility of conducting a larger blinded study.

Primary endpoint: To assess the effect of Lacosamide on CGRP-LI by detecting the difference in CGRP-LI level after three months of treatment in the two groups.

Secondary endpoint: The secondary safety endpoint was to assess the safety of lacosamide by assessing the different adverse effects through open‐ended patient interviews in the two groups.

The secondary efficacy endpoints evaluated the percentage of patients who achieved ≥ 50% reduction in the migraine monthly days (MMD) frequency compared to the baseline frequency and the percentage of patients who achieved pain freedom within 2 h in ≥ 4 of 5 attacks.

### Statistical analysis of the data

We used the IBM SPSS software package, version 20.0 (Armonk, NY: IBM Corp.), to analyze our data and base all efficacy analyses on the intention-to-treat principle. Both the primary and secondary outcomes underwent separate statistical analyses. Depending on their distribution, as determined by the Shapiro–Wilk test, we described numerical data as means S.D. or median and interquartile range (IQR). We also reported categorical data using numbers and percentages. The Mann–Whitney U test was used to compare the irregularly distributed numerical data, while Pearson's chi-square was utilized to correlate categorical data. In our study, there were all the data. All statistical analyses were two-sided, and differences with a P-value of less than 0.05 were considered statistically significant. To avoid type 1 statistical error in the analysis of secondary efficacy endpoints, we used correction for multiple comparisons, and secondary efficacy outcomes differences with an adjusted *P*-value of less than 0.025 were considered statistically significant.

## Results

**Fig. 1:**
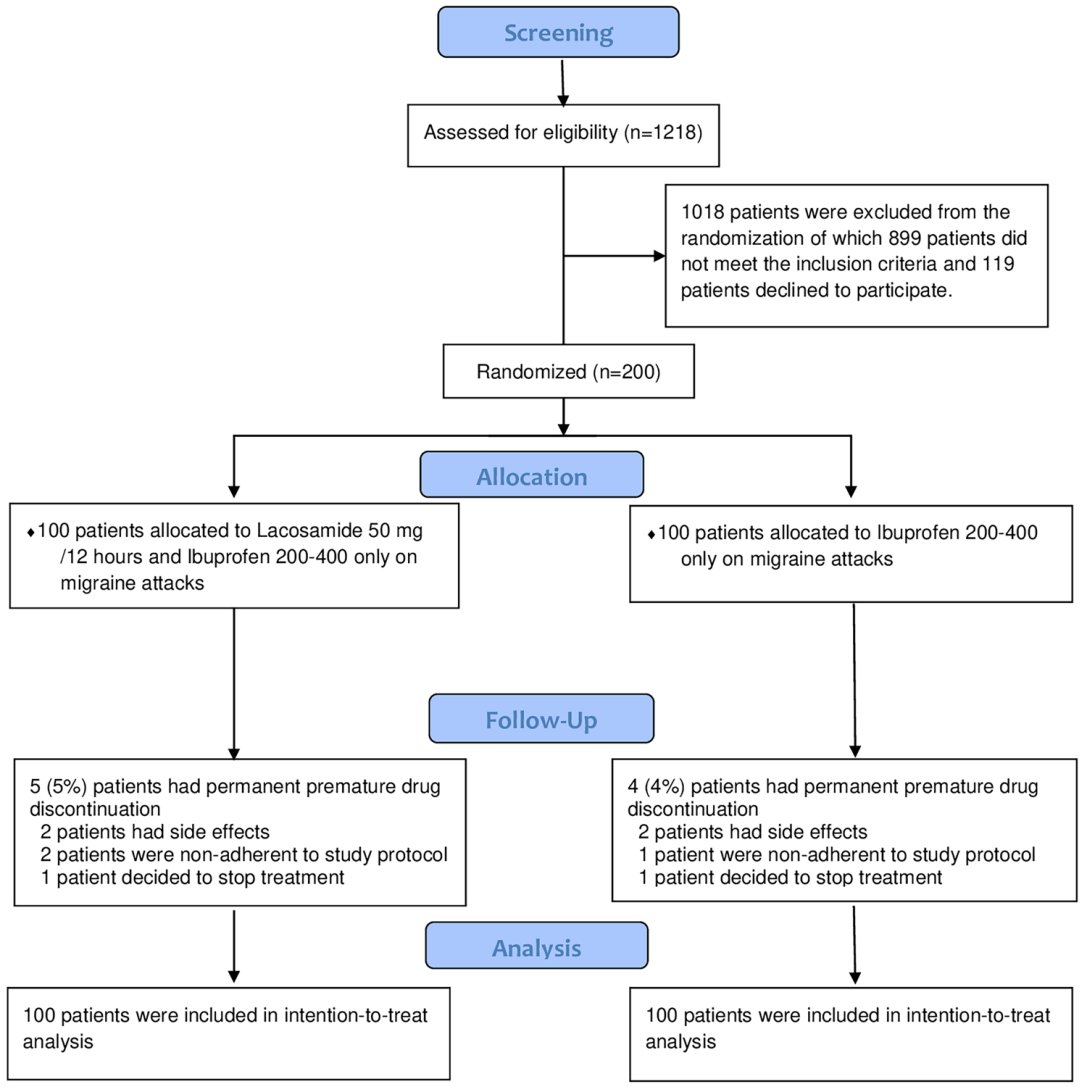
Study flow diagram

Overall, 1218 patients were screened for eligibility; 200 patients (80 males and 120 females) underwent randomization and were divided into two parallel groups. The lacosamide group consisted of 100 patients, and the control group consisted of 100 patients; 200 patients completed the pilot study during the 3-month follow-up period, as shown in (Fig. 1).

There were no statistically significant differences between the two study arms regarding the baseline characters, as shown in (Table 1).

**Table 1:** Baseline criteria of participants

Demographic data	Lacosamide arm (*n* = 100)	Control arm (*n* = 100)	*P*-value
Age (years)*			
10–20, no. (percentage)	12 (12%)	9 (9%)	0.80
21–30, no. (percentage)	43 (43%)	38 (38%)	
31–40, no. (percentage)	20 (20%)	24 (24%)	
41–50, no. (percentage)	12 (12%)	16 (16%)	
51–55, no. (percentage)	13 (13%)	13 (13%)	
Sex*			
Female, no. (percentage)	58.0 (58%)	62.0 (62%)	0.56
*Migraine characters†*			
Baseline MMD, Median (IQR)	7.0 (5.0–9.0)	7.0 (5.0–8.0)	0.52
Attack duration, Median (IQR)	6.0 (4.0–7.8)	6.0 (5.0–8.0)	0.68
Attack severity on Visual analog scale, Median (IQR)	6.0 (6.0–7.0)	6.0 (6.0–7.0)	0.69
Disease duration Median in months (IQR)	8.0 (7.0–10.0)	8.0 (6.0–10.0)	0.10
Migraine associated symptoms*			
Photophobia, no. (percentage)	82.0 (82.0%)	81.0 (81.0%)	0.90
Phonophobia, no. (percentage)	81.0 (81.0%)	77.0 (77.0%)	0.67
Nausea, no. (percentage)	79.0 (79.0%)	78.0(78%)	0.92
Dizziness, no. (percentage)	68.0 (68.0%)	69.0 (69.0%)	0.71
Vomiting, no. (percentage)	40.0 (40.0%)	31.0 (31.0%)	0.22
Migraine with aura, no. (percentage)	31 (31.0%)	25.0 (25.0%)	0.39
*Serum CGRP- IL levels†*			
Baseline Serum CGRP-IL value (Median, IQR) in episodic migraine patients	55.0 (47.5–66.0)	55.0 (47.0–66.0)	0.86

^†^Median (interquartile range: IQR), *Percentage, MMD: monthly migraine days, CGRP-LI: calcitonin gene-related peptide-like immunoreactivity

When we evaluated the effect of 3-month treatment on the serum level of CGRP-LI, we found that there was a statistically significant higher reduction in the serum CGRP-LI level in the lacosamide group compared with the control group with (*P* = 0.017), as shown in (Table 2).

**Table 2:** Association between Lacosamide and CGRP-LI level

CGRP-LI	Lacosamide arm (*n* = 100)	Control arm (*n* = 100)	*P*-value
Baseline serum CGRP-LI value (median, IQR)^†^	55.0 (47.5–66.0)	55.0 (47.0–66.0)	0.86
CGRP-LI After treatment (median, IQR)^†^	50.5 (44.3–59.1)	55.0 (47.0–66.0)	0.017*
Absolute difference in CGRP-LI (median, IQR)^†^	5.0 (4.0–5.8)	1.0 (0.5–1.2)	< 0.001*

^†^Median (interquartile range: IQR), *Statistically significant at *P*-value < 0.05, CGRP-LI: calcitonin gene-related peptide-like immunoreactivity

Regarding the analysis of the safety of treatment, we found that in the lacosamide group, 17(17%) patients had adverse effects; three patients had gastritis, four patients had a headache, two patients had vomiting, one patient had diarrhea, three patients had palpitation, and four patients had back pain, while in the control group 11(11%) patients had adverse effects, four patients had gastritis, two patients had a headache, one patient had vomiting, two patient had diarrhea, one patient had palpitation, and one patient had back pain, with no statistically significantly, as shown in (Table 3).

**Table 3:** Association between the treatment and main adverse effects

Adverse effects, no. (percentage)*	Lacosamide arm (*n* = 100)	Control arm (*n* = 100)	*P*-value
Gastritis	3.0 (3.0%)	4.0 (4.0%)	0.70
Headache	4.0 (4.0%)	2.0 (2.0%)	0.41
Vomiting	2.0 (2.0%)	1.0 (1%)	0.65
Diarrhea	1.0 (1%)	2.0 (2.0%)	0.31
Palpitation	3.0 (3.0%)	1.0 (1%)	0.31
Bach pain	4.0 (4.0%)	1.0 (1%)	0.17

*Percentage

Regarding the analysis of the clinical impacts of the treatment, we found that 42 (42%) patients in the lacosamide group and 22 (22%) patients in the control group achieved ≥ 50% reduction in the migraine monthly days (MMD) frequency with *P*-value 0.002, while 46 (46%) patients in the lacosamide arm and 30 (30%) patients in the control arm achieved pain freedom within two hours in ≥ 4 of 5 attacks with *P*-value 0.02, as shown in (Table 4).

**Table 4:** Association between Lacosamide and clinical outcomes

Secondary efficacy outcomes	Lacosamide arm (*n* = 100)	Control arm (*n* = 100)	*P*-value
Percentage of patients who achieved ≥ 50% reduction in MMD frequency compared to the baseline frequency*	42 (42.0%)	22.0 (22.0%)	0.002**
Percentage of patients who achieved pain freedom within two hours in ≥ 4 of 5 attacks*	46.0 (46.0%)	30.0 (30.0%)	0.020**

*Percentage, **Statistically significant at adjusted *P*-value < 0.025, MMD: migraine monthly days

## Discussion

The calcitonin gene-related peptide (CGRP) is one of the most significant peptides released during the migraine attack due to activation of the trigeminovascular system [2]. Lacosamide reacts with CRMP-2, and it prevents its phosphorylation leading to the inhibition of CGRP release in the trigeminal system [7], so we aimed in our trial to evaluate the effect of Lacosamide on CGRP-LI serum level and its efficacy, and safety in the treatment of episodic migraine patients.

We assessed the effects of lacosamide on CGRP and its efficacy, and safety in episodic migraine patients after three months only like many studies as Dakhale et al. [14] who compared the efficacy of daily sodium valproate 500 mg/day with propranolol SR 40 mg/day in 60 migraineurs after three months, and concluded that both sodium valproate and propranolol significantly reduced frequency, severity, and duration of migraine headache, but propranolol caused significantly greater reduction in the severity of headache compared to sodium valproate.

In our trial, 46 (46%) patients showed a reduction in CGRP-LI serum level after three months of treatment with lacosamide, while only 24 (24%) patients showed a reduction in CGRP-LI serum level after three months in the control group; also the absolute reduction in CGRP-LI serum level after three months of treatment was statistically significantly higher in migraine patients who received lacosamide and Ibuprofen compared with those who received ibuprofen alone.

Even though there has been no such study that investigated the effect of lacosamide on CGRP serum levels in migraine patients, our findings may be explained as lacosamide may reduce CGRP serum level by interaction with CRMP2 preventing its phosphorylation leading to the inhibition of CGRP [7].

Regarding the analysis of the safety of lacosamide, our study found that there was no statistically significant difference in adverse effects between the two groups; even though there has been no such study that investigated the safety of Lacosamide in episodic migraine patients, our findings are in line with results of Menachem et al. [18], Vossler et al. [19], and Bauer et al. [20] who stated that Lacosamide was well tolerated in epilepsy patients.

Regarding the analysis of the clinical impacts of lacosamide, our study found that regular use lacosamide was associated with increased percentage of patients who achieved ≥ 50% reduction in the migraine monthly days (MMD) frequency, and achieved pain freedom within two hours in ≥ 4 of 5 migraine attacks, our findings agreed with the findings of Yuan et al. [8] who stated that migraine patients showed a reduction in MMD after regular use of lacosamide, lacosamide might reduce MMD by enhancing the slow inactivation of voltage-gated sodium channels, interacting with the collapsin-response mediator protein 2 (CRMP-2), and reducing the CGRP level involved in neurotrophic pathways [21].

## Study limitations and conclusions

### Study limitations

Even though our results were positive, our pilot study had some drawbacks. First, the sample was relatively small as it was designed to determine the viability of a larger-scale trial that would be powered for both safety and efficacy; second, the trial was open-label, which might affect the study's internal validity by increasing the number of drop-out patients and underreporting of side effects; third, our study has no placebo group so we need a larger-scale double-blinded placebo-controlled trial to assess the lacosamide role in migraine.

### Conclusion

The daily use of lacosamide 50 mg Bid for three months in episodic migraine patients was associated with a significant reduction in serum CGRP-LI, better clinical outcomes regarding the frequency and duration of migraine attacks, and was well tolerated by patients, which may indicate a possible role of lacosamide in episodic migraine management. These results were derived from an open-label pilot study that needed to be thoroughly investigated by a large-scale, randomized, double-blinded, placebo-controlled study.

## Data Availability

The datasets generated and analyzed during the current study are not publicly available due to the ethical regulations of our university. However, they are available from the corresponding author (Mohamed G. Zeinhom) on reasonable request.

## Abbreviations

CGRP-LI: Calcitonin gene-related peptide-like immunoreactivity
CRMP-2: Collapsin response mediator protein 2
ECG: Electrocardiography
HbA1C: Hemoglobin A1c
ICHD-3: The international classification of headache disorders
IQR: Inter-quartile range
MMD: Migraine monthly days
MRI: Magnetic resonance imaging
SD: Standard deviation
VGSCs: Voltage-gated sodium channels
